# Supplementary material for: Ground state selection under pressure in the quantum pyrochlore magnet Yb2Ti2O7
Source: Nat Commun. 2017 Mar 15;8:14810. doi: 10.1038/ncomms14810 (PMC5355945; doi:10.1038/ncomms14810)
Supplement: Supplementary Information — Supplementary figures, supplementary notes and supplementary references. [file ncomms14810-s1.pdf]

Supplementary Note 1 background subtraction for μSR measurements

The relaxation of both cells was measured without any sample from 2 K down to 0.245 K. The relaxation of each cell (R_{cell}) was fitted separately and subsequently subtracted from the whole signal (pressure cell + sample) in order to extract the relaxation due to the sample only (R_{sample}). The total signal in zero field $R_{\text{zf}}(t)$ is fitted to the following general function:

$$A_{\text{tot}} R_{\text{zf}}(t) = A_{\text{tot}} \left[f_{\text{cell}} R_{\text{cell}}(t) + (1 - f_{\text{cell}}) R_{\text{sample}}(t) \right] \tag{1}$$

The total asymmetry A_{tot} is determined by a weak transverse-field measurement above the magnetic transition. At low temperature, the polarization relative to the sample can be easily distinguished from the pressure cell signal due to the difference of relaxation, and we estimate $f_{\text{cell}} = 0.545$, a typical value for experiments using such double-wall cells. This fraction was then held fixed for all subsequent fits. We detail below the measurements of the relaxation of each pressure cell, $R_{\text{cell}}(t)$.

Supplementary Fig. 1a) shows the relaxation obtained for the pressure cell (1) only, at 2 K and 0.245 K. These relaxations could be well fitted to a dynamical Kubo-Toyabe (DKT) function:

$$R^{(1)}_{\text{cell}}(t) = (1 - B)\text{DKT}(\nu, \sigma, \Gamma, t) + B \tag{2}$$

with $\nu = 0$ (zero-field), σ the width of the Gaussian distribution, Γ the hopping rate and the background value $B = 0.035$. σ and Γ are found to smoothly evolve from 2 K ($\sigma = 0.361(2)$ μs^{-1}, $\Gamma = 0.20(2)$ μs^{-1}) to 0.245 K ($\sigma = 0.500(4)$ μs^{-1}, $\Gamma = 0.63(3)$ μs^{-1}) and we thus used interpolated values for the subtraction at intermediate measured temperatures assuming an exponential

variation with temperature. Supplementary Fig. 1b) shows a typical measurement with the loaded pressure cell at $P = 19.7$ kbar, i.e. with muons stopping inside the pressure cell and the sample and Supplementary Fig. 1c) shows the result of the subtraction at 0.245 K fitted to the following phenomenological function:

$$R_{\text{sample}}(t) = f\left(\frac{2}{3}e^{-\Delta^2 t^2/2} + \frac{1}{3}e^{-\lambda t}\right) + (1-f)e^{-\lambda t} \tag{3}$$

with f the volume magnetic fraction, Δ the Gaussian width of the static field distribution, and λ the paramagnetic relaxation rate used for both the 1/3-tail and the paramagnetic component, for simplicity. The departure from the traditional static or dynamic Gaussian Kubo-Toyabe function was already observed and studied in details in Supplementary Ref.[2–4].

Supplementary Fig. 2a) shows the relaxation obtained for the pressure cell (2) only, at 1.8 K and 0.245 K. These relaxations could be well fitted to a single exponential form:

$$R^{(2)}_{\text{cell}}(t) = e^{-\lambda_c t} \tag{4}$$

with λ_c the exponential relaxation rate. λ_c was found to smoothly evolve from 1.8 K ($\lambda_c = 0.077\ \mu\text{s}^{-1}$) to 0.245 K ($\lambda_c = 0.134\ \mu\text{s}^{-1}$) and we thus used interpolated values for the subtraction at intermediate measured temperatures assuming an exponential variation with temperature. Supplementary Fig. 2b) shows a typical measurement with the loaded pressure cell at $P = 25$ kbar, i.e. with muons stopping inside the pressure cell and the sample and Supplementary Fig. 2c) shows the result of the subtraction at 0.245 K fitted to the phenomenological function (equation (3) in Supplementary Note 1).

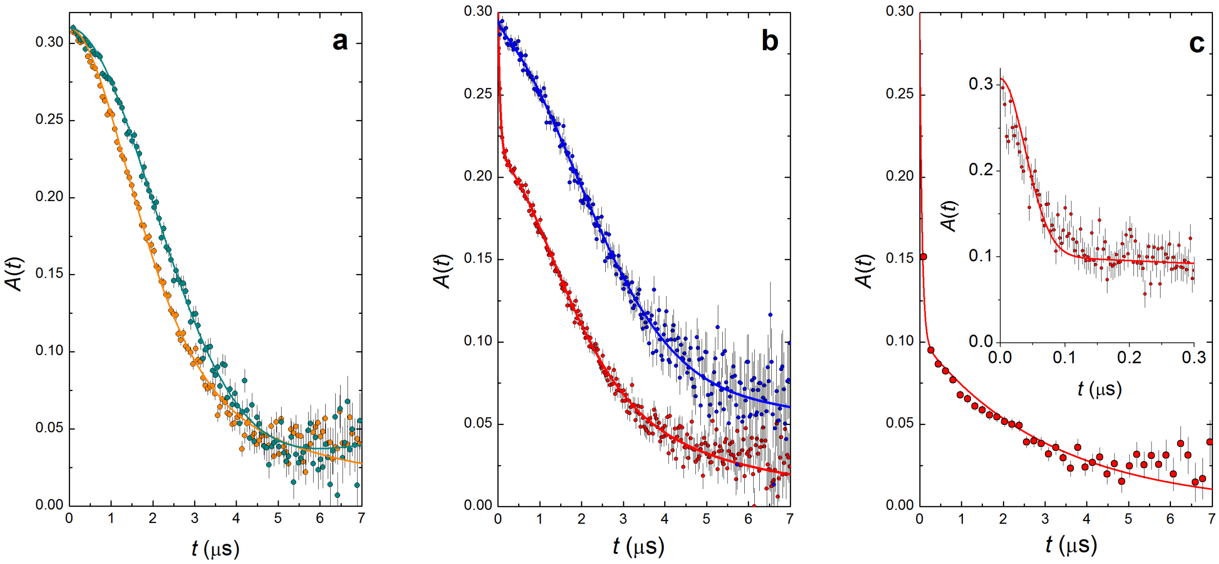

Supplementary figure 1: **Background subtraction for the pressure cell (1)**. $A(t) = A_{\text{tot}}R_{\text{zf}}(t)$ is the measured asymmetry as a function of time t. a) Relaxation of the pressure cell (1) only $[f_{\text{cell}} = 1]$, measured at 0.245 K (orange) and 2 K (dark cyan). Lines are fits to the equation (2) in Supplementary Note 1. b) Relaxation measured above (1.5 K) and below (0.245 K) the magnetic transition under an applied pressure $P = 19.7$ kbar for a loaded pressure cell $[f_{\text{cell}} = 0.545]$. Lines are fit to to the equations (1), (2) and (3) in Supplementary Note 1. c) Relaxation relative to the sample only after subtraction of the pressure cell signal, measured at 0.245 K. Line is a fit to the equation (3) in Supplementary Note 1. Inset: Zoom at short times. The error bars of the μSR relaxation data are of statistical origin and correspond to the square root of the total number of detected positrons resulting from muon decays.

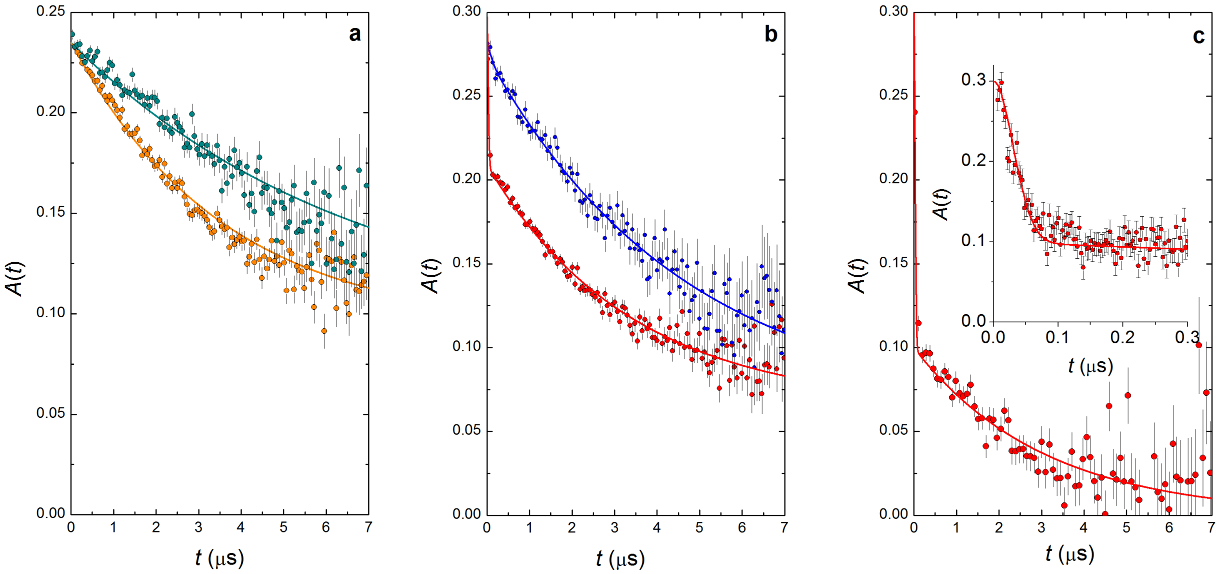

Supplementary figure 2: **Background subtraction for the pressure cell (2)**. $A(t) = A_{\rm tot}R_{\rm zf}(t)$ is the measured asymmetry as a function of time t. a) Relaxation of the pressure cell (2) only $[f_{\rm cell} = 1]$, measured at 0.245 K (orange) and 1.8 K (dark cyan). Lines are fits to the equation (4) in Supplementary Note 1. b) Relaxation measured above (0.86 K, blue) and below (0.245 K, red) the magnetic transition under an applied pressure $P = 25$ kbar for a loaded pressure cell $[f_{\rm cell} = 0.545]$. Lines are fit to the equations (1),(4) and (3) in Supplementary Note 1. c) Relaxation relative to the sample only after subtraction of the pressure cell signal, measured at 0.245 K. Line is a fit to the equation (3) in Supplementary Note 1. Inset: Zoom at short times. The error bars of the μSR relaxation data are of statistical origin and correspond to the square root of the total number of detected positrons resulting from muon decays.

Supplementary Note 2 neutron diffraction

A typical diffraction pattern obtained for $T = 0.800$ K is shown in Supplementary Fig. 3 for which the assignment of some of the structural Bragg peaks of $Yb_2Ti_2O_7$ and the NaCl are indicated. Contributions to the scattering coming from the cryostat as well as the pressure cell create a substantial background, especially in the region of Q going from 1.25 Å^{-1} to 1.75 Å^{-1}, and this region is thus excluded from all refinements. The resulting lattice parameter for NaCl is 5.54 Å. Using a calibration table for the lattice parameter of NaCl as a function of the applied pressure[5], we deduced the pressure in the cell to reach 11±2 kbar. The structural refinement of $Yb_2Ti_2O_7$ at $T = 0.800$ K was assumed to be of nuclear origin only and is consistent with a slightly smaller lattice parameter as well as a lower axial distortion of the eight oxygens surrounding the Yb^{3+} atoms than at ambient pressure. However, the large value of our uncertainty precludes any quantitative analysis.

In addition to $T = 0.800$ K, the diffraction pattern of $Yb_2Ti_2O_7$ has also been collected for T =0.100, 0.200, 0.250, 0.300 and 0.400 K. Fig. 4a) of the main text shows the resulting magnetic diffraction pattern at low temperature where the 0.800 K data set has been subtracted as a background. For T =0.100 K, we observed a clean subtraction of the background signal but an excess of scattering is seen at the (111), (113), (222) and (004) Bragg peaks position of $Yb_2Ti_2O_7$. This purely magnetic scattering was refined using a fixed scale factor which had been determined by the purely nuclear refinement of the paramagnetic 800 mK dataset. As every magnetic Bragg peak is located on a nuclear allowed position of $Yb_2Ti_2O_7$, we assumed a k =0 magnetic structure.

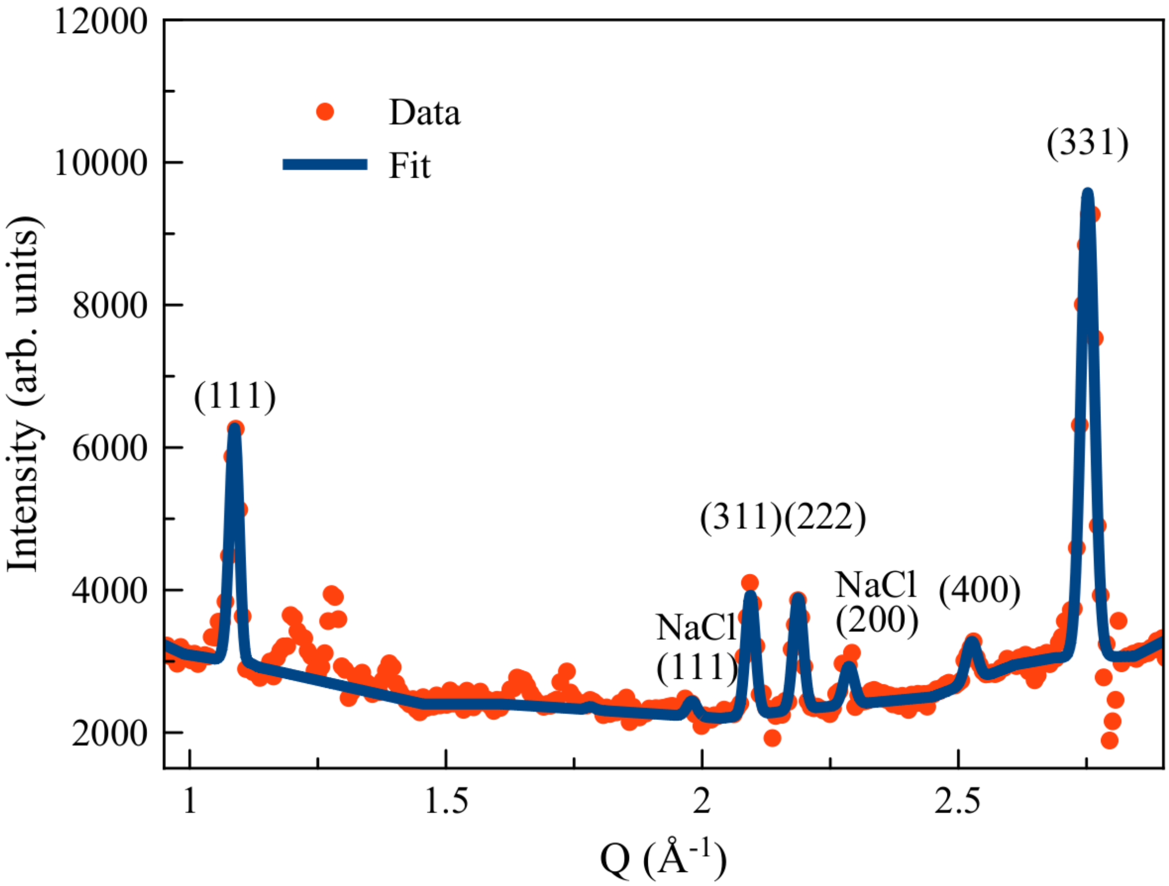

Supplementary figure 3: **Structural refinement of the neutron diffraction pattern.** Diffraction pattern measured at $T = 0.800$ K (red points) and structural refinement for both the $Yb_2Ti_2O_7$ and the NaCl phases (blue line). The region of Q from 1.125 Å^{-1} to 1.75 Å^{-1} is polluted from spurious contributions coming from the cryostat and the pressure cell, and thus excluded from the refinement. The refined value of the lattice parameter of NaCl is used to determine the applied pressure of 11±2 kbar.

The possible irreducible representation for the $16d$ site of the $Fd\bar{3}m$ pyrochlore lattice are Γ_3, Γ_5, Γ_7 and Γ_9. The Γ_9 representation manifold gives the best agreement with our data and the resulting magnetic moment is 0.33(5) μ_B at $T = 0.100$ K. The refined spin structure consists of all spins pointing almost fully along the [100] direction but slightly tilted towards the [111] local direction by $5 \pm 4°$. Finally, the same refinement has been performed for all other measured temperatures and a decrease of the resulting magnetic moment is observed up to 0.400 K for which no extra scattering is observed (Fig. 4b) of the main text).

Supplementary references

1. Khasanov, R., *et al*. High pressure research using muons at the Paul Scherrer Institute. *High Pressure Research* **36**, 140-166 (2016).

2. Hodges, J. A. *et al*. First-order transition in the spin dynamics of geometrically frustrated $Yb_2Ti_2O_7$. *Phys. Rev. Lett.* **88,** 077204 (2002).

3. Yaouanc, A., Maisuradze, A. & Dalmas de Réotier, P. Influence of short-range spin correlations on the μSR polarization functions in the slow dynamic limit: application to the quantum spin-liquid system $Yb_2Ti_2O_7$. *Phys. Rev. B* **87,** 134405 (2013).

4. Maisuradze, A., *et al*. Anomalously slow spin dynamics and short-range correlations in the quantum spin ice systems $Yb_2Ti_2O_7$ and $Yb_2Sn_2O_7$. *Phys. Rev. B* **92**, 094424 (2015).

5. Decker, D. L. Equation of state of NaCl and its use as a pressure gauge in high-pressure research. *J. Appl. Phys.* **36**, 157 (1965).

6. Rodriguez-Carvajal, J. Recent advances in magnetic structure determination by neutron powder diffraction. *Physica B : Condensed Matter* **192**, 55 (1993).
